# PARTICIPATION IN DIVERSE SOCIAL ACTIVITIES IS ASSOCIATED WITH LOW MORTALITY RISK

**DOI:** 10.1093/geroni/igad104.0924

**Published:** 2023-12-21

**Authors:** Sangha Jeon, Nicholas Turiano, Susan Charles

**Affiliations:** University of California, Irvine, Irvine, California, United States; West Virginia University, Morgantown, West Virginia, United States; University of California, Irvine, Irvine, California, United States

## Abstract

Social activity is often associated with better physical health outcomes. Yet, less is known about specific qualities of social activity beyond its frequency, such as whether engagement in diverse social activities (social activity variety) is important for health. The current study assessed whether social activity variety, which was calculated as the number of different types of social activity, relates to mortality risk beyond non-social activity variety and how its relationship varies depending on age groups. Using data from the Health and Retirement Study (HRS), we included 5,559 adults who completed a questionnaire on social activity participation in 2008 and whose mortality status was recorded in 2019. We also examined whether age was related to the relationship between social activity variety and mortality risk. Cox proportional hazard model analyses revealed that those with higher social activity variety were more likely to survive over the following 11 year than those with low social activity variety. This result remains robust when adjusting for health status and non-social activity variety. The association of social activity variety with mortality risk is significant across all age, but it is stronger in older adults compared to young adults. Findings suggest that social activity variety is beneficial for longevity beyond non-social activities and health status across adulthood.

